# Distortion correction of MR data in whole-body small animal PET-MR using 3D thin-plate splines

**DOI:** 10.1186/2197-7364-1-S1-A89

**Published:** 2014-07-29

**Authors:** Lynn Frohwein, Verena Hoerr, Cornelius Faber, Klaus Schäfers

**Affiliations:** European Institute for Molecular Imaging (EIMI) – University of Muenster, Kragujevac, Germany; Department of Clinical Radiology, University Hospital of Muenster, Kragujevac, Germany

MR imaging suffers from geometric distortion caused by gradient nonlinearities at the edges of the gradients in large Field-of-Views (FOV). Therefore, the fusion of PET and MR data can be deficient. This can be an important aspect in whole-body imaging as well as for volumetric measurements. In this work a marker-based method is presented to increase the accuracy of the fusion of PET and MR data by correcting for these geometric distortions through 3D thin-plate splines [1].

For the determination of the geometric distortion in the MR data a customized phantom (112 mm in length) with a 3-dimensional grid of 575 (5x5x23) sphere-shaped fillable control points has been developed. Phantom MR data is acquired with two standard MR sequences (FLASH, RARE) on a BioSpec 94/20 (Bruker). Additionally, a 20 minute [F18]-FDG PET scan of the phantom is performed on a quadHIDAC-PET-scanner (Oxford Positron Systems). The positions of the control points are determined semiautomatically on both MR (distorted grid) and PET data (reference grid). The transformation between both modalities is calculated with 3D thin-plate splines (3D TPS). The calculated transformation is then applied to whole-body mouse data, which is acquired with the same scan parameters as used in the phantom scans.

**Figure 1:**
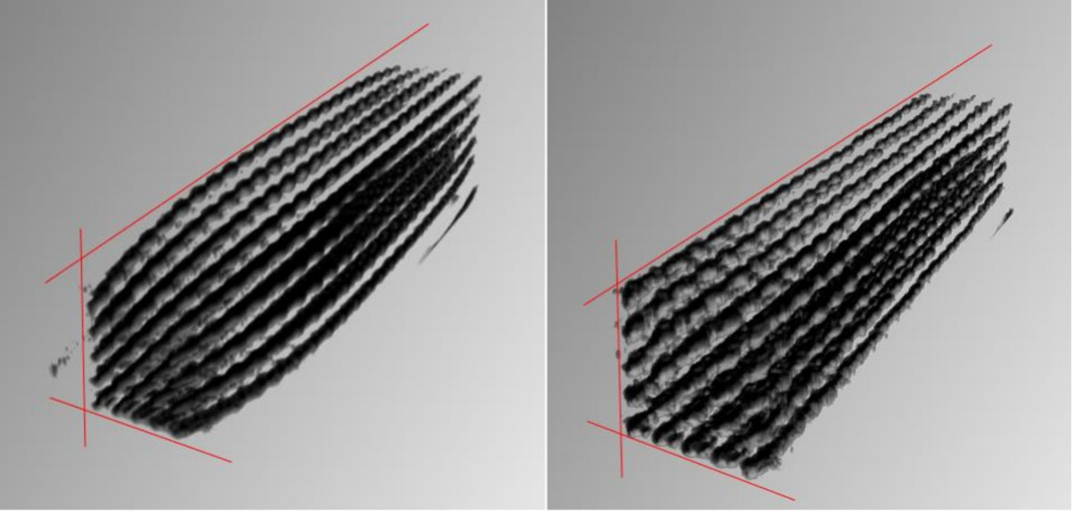
3D rendered MR data of the phantom used for distortion correction. Left: distorted MR data with bowed lines, Right: straightened lines after distortion-correction

**Figure 2:**
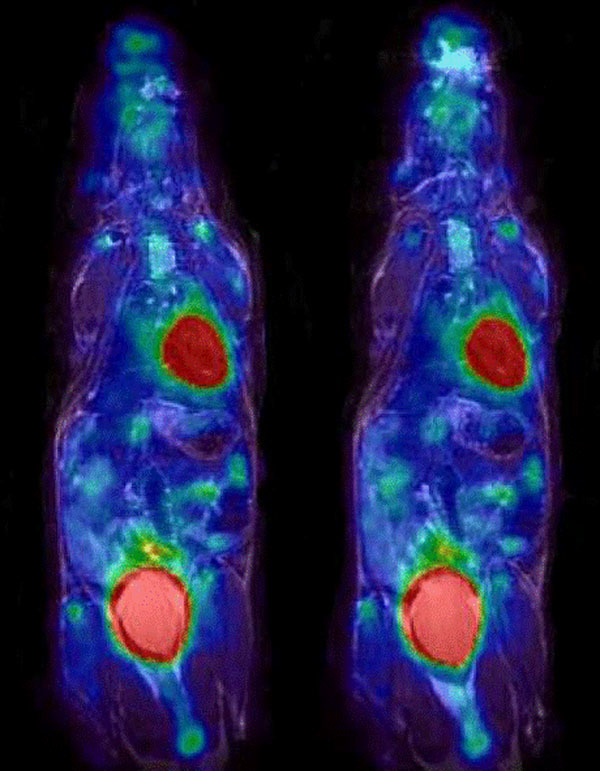
Left: distorted MR data overlaid with PET data. In particular, the head region of the mouse is poorly congruent with PET data. Right: distortion- corrected MR data overlaid with PET data. The head region now shows better congruency.

Lines formed by the control points along sagittal planes, which were distorted in a bow-like manner by gradient nonlinearities in the uncorrected MR data (max. deviation: about 3 mm), are straightened after correction (Figure 1). After the distortion-correction PET and MR data can thus be fused more congruently than without distortion-correction (Figure 2).

A phantom has been developed which allows the determination of geometric distortions in MR data. Furthermore, a method to correct for geometric distortion based on the transformation information calculated from the phantom data with 3D TPS has been implemented.
